# Prevalence, Diagnosis, and Vaccination Situation of Animal Chlamydiosis in China

**DOI:** 10.3389/fvets.2018.00088

**Published:** 2018-05-30

**Authors:** Jizhang Zhou, Zhaocai Li, Zhongzi Lou, Yuanyuan Fei

**Affiliations:** State Key Laboratory of Veterinary Etiological Biology, Lanzhou Veterinary Research Institute, Chinese Academy of Agricultural Sciences, Lanzhou, China

**Keywords:** prevalence, diagnosis, vaccine, *Chlamydia*, China

## Abstract

Since the first case of* Chlamydia* infection in duck had been reported in 1956 and the first case from domestic animal had been reported in 1979 in China, the chlamydia prevalence in China was heavily according to the published data. The* Chlamydi*a in avian prevalence has been reported at least 11 provinces, *Chlamydia* in sheep and goats at least 11 provinces, in swine at least 15 provinces, in cows at least 13 provinces and in yaks at least 5 provinces with result of IHA detection. Different diagnostic method such as CFT, ELISA and ABC-ELISA (avidin-biotin-complex ELISA) had been established besides IHA. The inactivated vaccines have been developed with isolated strains from sheep, goats, swine and cows. These inactivated vaccines have been used since 1980s and *Chlamydia* prevalence in China has been successfully controlled in domestic animal. However, the inactivated vaccines of Chlamydia isolated from avian species have not been successful, although a series of experimental vaccine have been done. Due to the unsustainable eradication plan of *Chlamydia* in China, sporadic outbreak in animal would happen if the vaccinations were suspended and economy lose in some farmers. Although Chlamydia prevalence in China has a long history, however, almost all published studies are in Chinese, which, in some degree, blocked scientists in other countries to understand the prevalence situation and control measures of *Chlamydia* in China.

## Introduction

Chlamydiae, zoonotic and obligate intracellular Gram-negative bacteria, have a worldwide distribution and cause a wide range of diseases in human hosts, livestock, companion animals, wildlife, exotic species (1), and poultry (2). Chlamydiosis, a disease caused by *Chlamydia,* has been found in many countries around the world where sheep-rearing is practiced, ranging from Europe to Africa, North America (3), and Asia (4). These include France (5), Poland (6), Spain (7), Australia (8), the United Kingdom (9), Ireland (10), China (11) and Switerland (12).

Vaccination is one of the best ways to control Chlamydiosis. Formalin-inactivated, egg-grown vaccine of *Chlamydia abortus* was developed (13) in the U. K. The inactivated vaccine of *C. abortus* was used in sheep although the efficacy of the vaccine against EAE was unstisfatory (14). After the first reported cases of avian *Chlamydia* in China in 1959 (15) and reports of infection in domestic animals in Qinghai province in 1981 (4), China began developing inactivated vaccines using different isolates from sheep, swine, and cow, which successfully controlled the prevalence of *Chlamydia* in China. However, almost all these achievements were reported only in Chinese, rather than in English, which presents a challenge for scientists all over the world to understand the situation of *Chlamydia* prevalence and control measures in China.

## *Chlamydia* Prevalence

Following the first report of *Chlamydiosis* in China from a duck in Beijing in 1959 (15), prevalence of avian *Chlamydia* has been reported in 10 provinces, including Beijing, Tianjin, Jiangsu, and Guangdong due to lack of effective vaccines (16).

**Figure 1:**
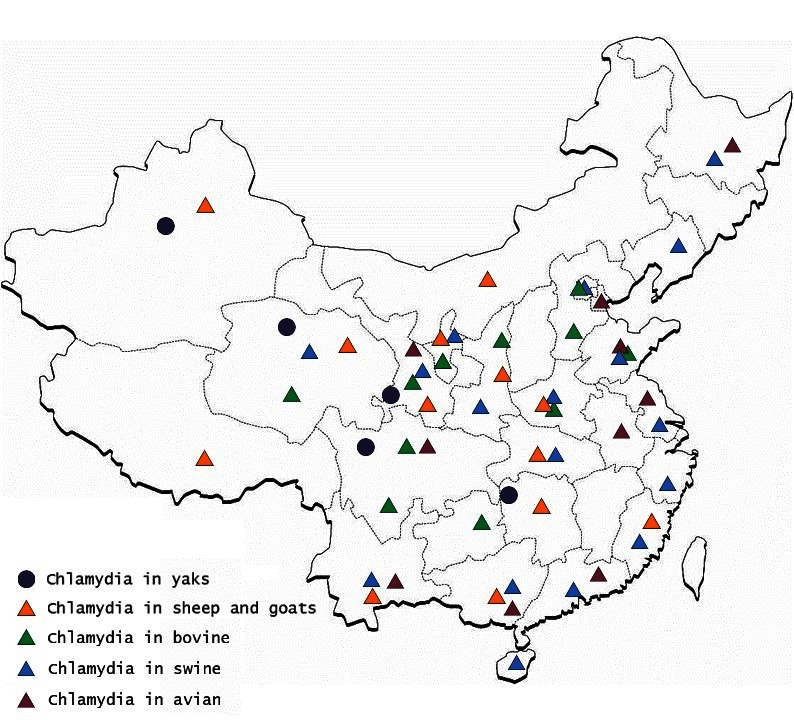
Distribution of *Chlamydia* prevalence in different animals and areas in China.

The first instance of livestock *Chlamydiosis* was found in ruminants in the Qinghai province by scientists from the Lanzhou Veterinary Research institute (LVRI), Chinese Academy of Agricultural Sciences (CAAS), in 1979 (17). *Chlamydia* was detected in sheep and goats in at least 11 provinces, in swine in 15 provinces, in bovines in 13 provinces, and in yaks in 5 provinces, using the indirect hemagglutination assay (IHA) test; this prevalence if the disease caused a huge economic loss in China (Figure 1). Although the seropositive rate was high in different animals in China, similar seropositive rates existed in other countries. For example, a German study reported *Chlamydia* antigen prevalence in sheep to range between 5 and 71% (18) and a Polish study reported the prevalence of *Chlamydial* infection in birds in Europe to range between 6.3 and 19.2% (19). There was uncertainty about the accuracy of the results of *Chlamydia* detected in animals using the IHA technique in China. However, compared with the results from other countries, which used McCoy cell culture isolation, the IHA technique may reflect the real prevalence of *Chlamydia* in China.

Using IHA, *Chlamydia* was found to be prevalent in chicken, ducks, pigeons, geese, and other avian species in 11 provinces, with seropositive rates of 10 to 60% in last three decades (20–22) (Table 1). Likewise, *Chlamydia* was prevalent in cows, yaks, sheep, goats, and pigs with seropositive rates of 2–40% in goats and sheep (4, 33–38) (Table 2), 5–53% in swine (50–55) (Table 3), 3–35% in bovine (67–71) (Table 4), and 2–30% in yak (11, 76–79) (Table 5). The disease was found in almost the entire country through IHA detection.

**Table 1:** Avian *Chlamydia* seroprevalence in China.

**Year**	**Positive rate**	**Area**	**Reference (published in Chinese)**
**1991**	21.8%	Shandong	Wu *et al.* (1990:61) (23)
**1988–1989**	39.2%	Sichuan	Xu *et al.* (1991:18–19) (24)
**1991**	26.91%	Gansu	Wang *et al.* (1991:18) (25)
**1994**	20–40%	Jiangsu	Yu *et al.* (1994:13–15) (20)
**1994**	22.7%	Yunnan	Wang *et al.* (1997:10–12) (26)
**1998**	5.1%	Heilongjiang	Jiang *et al.* (1998:25–6) (27)
**2001**	20.66%	Guangxi	Liu *et al.* (2001:13) (28)
**2003**	10–30%	Beijing Tianjin	Shi *et al.* (2003:217–21) (21)
**2003**	59.9%	Guangdong	Zhang *et al.* (2003:29) (29)
**2012**	20–45.4%	Anhui	Ou *et al.* (2012:61-3) (30)
**2013**	3.9%	Qinghai	Ma *et al.* (2013:213–5) (31)
**2012–2014**	36.97%	Tianjin	Zhu *et al.* (2016:148–50) (32)
**2016**	43.2%	Sichuan	Ouyang *et al.* (2016:46–51) (22)

**Table 2:** Sheep and goat *Chlamydia* seroprevalence in China.

**Year**	**Positive rate**	**Area**	**Reference (published in Chinese)**
**1981**		Qinghai	Yang *et al.* (1981:13–15) (4)
**1987–1989**	6.9%	Zhejiang	Wang *et al*.(1990:11) (39)
**1988–1989**	2.95%	Hubei	Zhang *et al.* (1992:34) (40)
**1998**	5.78%	Hunan	Qiu et al.(1998:3-5) (41)
**1996–1998**	1.98%	Guangxi	Wu *et al.* (2000:41) (36)
**1991–1995**	26.12%	Yunan	Wang *et al.* (2000:465–6) (42)
**2003**	19.3%	Ningxia	Bao *et al.* (2003:13–14) (43)
**2009**	7.57%	Neimeng	Wang *et al.* (2009:154) (44)
**2010–2011**	36.12%	Guizhou	Hong *et al.* (2012:127–9) (37)
**2012**	1.4%	Xinjiang	Lei *et al.* (2012:28-9) (45)
**2013**	20.9%	Xizang	Huang *et al.* (2013:243–5) (in English) (46)
**2014**	40.3%	Qinghai	Zhang* et al.* (2014:38–9) (47)
**2014**	60%	Hubei	Cheng *et al.* (2015:472–4) (48)
**2014**	42.86%	Anhui	Cheng *et al.* (2015:472–4) (48)
**2014**	52.75%	Shandong	Cheng *et al.* (2015:472–4) (48)
**2014**	40.13%	Xinjiang	Cheng *et al.* (2015:472–4) (48)
**2014**	51.91%	Jilin	Cheng *et al.* (2015:472–4) (48)
**2014**	30.56%	Sichuan	Cheng *et al.* (2015:472–4) (48)
**2014**	67.74%	Ningxia	Cheng *et al.* (2015:472–4) (48)
**2014**	90%	Gansu	Cheng *et al.* (2015:472–4) (48)
**2012–2015**	13.09%	Qinghai	Su.(2016:29-30) (35)
**2015–2016**	12.5%	Henan	Gao *et al.* (2017:66–9) (49)
**2013–2017**	11.87%	Qinghai	Zha *et al.* (2017:60–1) (38)

**Table 3:** Swine *Chlamydia* seroprevalence in China.

**Year**	**Positive rate**	**Area**	**Reference (published in Chinese)**
**1983–1984**	29.72%	Hubei	Jiang *et al.* (1985:32–4) (50)
**1985**	33.3%	Qinghai	Diao *et al.* (1990:21–2) (56)
**1991**	20.4%	Guangxi	Yi *et al.* (1991:6–9) (55)
**1997**	13.65%	Shandong	Ji *et al.* (2003:39) (57)
**1998–2000**	34.91%	Gansu	Gao *et al.* (2001:13–14) (58)
**2003**	2.3%	Henan	Lang *et al.* (2004:29) (59)
**2003**	5.16%	Liaonin	Wang (2004:26) (60)
**2005**	49.49%	Hainan	Suo *et al.* (2005:31–2) (51)
**2005**	41.41%	Shanghai	Jin *et al.* (2005:23) (61)
**2005**	6.82%	Zhejiang	Jin *et al.* (2005:23) (61)
**2004–2006**	14.97%	Guangdong	Zhu *et al.* (2007:26–7) (62)
**2006–2007**	27.71%	Fujian	Zhou *et al*. (2008:30–5) (63)
**2011**	18.5%	Yunnan	Bi *et al.* (2011:134–6) (52)
**2012**	7.6%	Shaanxi	Wang *et al.* (2013:9–10) (64)
**2013**	53.30%	Qinghai	Ma *et al.* (2013:213–5) (31)
**2014**	58.59%	Jiangxi	Jang* et al.* (2014:27–28) (65)
**2014**	57.0%	Qinghai	Zhang* et al.* (2014:38–9) (47)
**2014**	18.88%	Yunnan	Li* et al.* (2014:29–30) (66)
**2015**	18.4%	Yunnan	Su *et al.* (2015:155–6) (53)
**2013–2015**	11.3%	Henan	Ma* et al.* (2016:119–22) (54)

**Table 4:** Bovine *Chlamydia* seroprevalence in China.

**Time**	**Positive rate**	**Area**	**Published in original journal**
**1988**	26.81%	Hubei	Yang *et al.* (1988:5–6) (72)
**1997–1998**	16.13%	Shandong	Zhou *et al.* (2000:14–15) (67)
**1997–1998**	23.1%	Hebei	Zhou *et al.* (2000:14–15) (67)
**1997–1998**	43.18%	Shaanxi	Zhou *et al.* (2000:14–15) (67)
**1997–1998**	25.71%	Henan	Zhou *et al.* (2000:14–15) (67)
**1997–1998**	20.68%	Gansu	Zhou *et al.* (2000:14–15) (67)
**1997–1998**	25.6%	Sichuan	Zhou *et al.* (2000:14–15) (67)
**1997–1998**	16.8%	Ningxia	Zhou *et al.* (2000:14–15) (67)
**1997–1998**	15.49%	Qinghai	Zhou *et al.* (2000:14–15) (67)
**2012**	24.14%	Ningxia	Xie *et al.* (2012:102–4) (68)
**2010–2011**	2.86%	Guizhou	Hong *et al.* (2012:127–9) (37)
**2013**	21.3%	Chongqing	Huo (2013:67) (73)
**2013**	26.4%	Qinghai	Ma *et al.* (2013: 213-5) (31)
**2013**	17.71%	Gansu	Tan* et al.* (2015:1283–7) (74)
**2013**	38.97%	Ningxia	Tan* et al.* (2015:1283–7) (74)
**2014**	36.8%	Qinghai	Zhang* et al.* (2014:38–9) (47)
**2012–2015**	8.25%	Qinghai	Su.(2016:29–30) (35)
**2015**	26.31%	Qinghai	Wang *et al.* (2016:16–17) (69)
**2015**	37.43%	Henan	Li* et al.* (2017:22–4) (71)
**2015–2016**	1.75%	Qinghai	Chen* et al.* (2017:33–5) (75)
**2013–2017**	9.13%	Qinghai	Zha *et al.* (2017:60–1) (38)

**Table 5:** Yak *Chlamydia* seroprevalence in China.

**Year**	**Positive rate**	**Area**	**Reference (published in Chinese)**
**1988**	29.0%	Qinghai	Shuai* et al.* (1988:76–81) (76)
**1993**	21.03%	Qinghai	Dong *et al.* (1993:25) (80)
**1996**	2.1%	Xinjiang	Wang* et al.* (1996:46) (77)
**2000**	20.69%	Gansu	Zhou* et al.* (2000:14–15) (67)
**2000**	15.49%	Qinghai	Zhou* et al.* (2000:14–15) (67)
**2000**	25.6%	Sichuan	Zhou* et al.* (2000:14–15) (67)
**2004**	19.23%	Qinghai	Ma *et al.* (2004:14) (81)
**2009**	9.81%	Qinghai	Zhang *et al.* (2009:14–15) (82)
**2010**	17.39%	Qinghai	Hou(2011:10) (78)
**2010–2012**	2.8%	Qinghai	Kong *et al.* (2012:51–51) (83)
**2012**	4.13%	Qinghai	Li *et al.* (2013:126) (84)
**2013**	27.7%	Qinghai	Xie(2013:33) (85)
**2012–2013**	15.9%	Gansu	Qin(2015:8) (in English) (79)
**2014**	25.08%	Gansu	Yin(2014:281–285) (86)
**2009–2014**	7.68%	Qinghai	Fu *et al.* (2016:50–51) (87)
**2015**	23.81%	Qinghai	Li* et al.* (2015:1–6) (in English) (11)

## Diagnostic Method

Before 1984, antibodies to *Chlamydia* were detected by using the complement fixation (CF) method in China. However, this technique was cumbersome and time consuming (88). Thus, a new technique, IHA, was developed. The IHA technique had a higher sensitivity and specificity than CF, and has been used to determine the prevalence of* Chlamydia* in domestic animals in China since the 1980s (88). It has also been used as a high throughput method of seroprevalence detection. Positive results are detectable within 2 h, but false positive and negative readings are possible, since scoring is subjective to the researcher’s observation (88). Besides IHA, enzyme linked immunosorbent assay (ELISA) (89) and avidin-biotin-complex ELISA (ABC-ELISA) (90) techniques were developed. Although the ABC-ELISA and ELISA methods have higher sensitivity than the IHA method, they have not been used in clinical studies due to high cost.

For pathogen diagnosis, chicken egg isolation and Giemsa staining were combined to detect *Chlamydia*. The yolk sac membranes from dead chicken embryos were spread on slides and fixed with methanol or through heating, and Giemsa stain was used to stain them for half an hour or overnight. *Chlamydia* elementary body (EB) and inclusion were detected by light microscopy (91).

The PCR and real-time PCR tests, although highly sensitive and used to detect *Chlamydia* in different animals in other countries (92–96) have seldom been used on a large scale due to high cost. The *omp1* gene, which is very conservative, was used as a target gene to detect *Chlamydia* in different animals when IHA results were ambiguous (16, 97). In China, the PCR-RFLP method was developed and used only to identify *Chlamydia* species isolated from animals (11), while recombinase polymerase amplification (RPA) was used to identify *C. abortus* (98). However, IHA is considered as a simple, safe, and reliable means of testing *C. abortus* antibodies, and has been employed in previous serological investigations (79, 99).

## Prevention and control of Chlamydiosis

### Vaccination

Vaccination is one of the most important methods of disease prevention in animals. The inactivated vaccine of *C. abortus* plays a huge role to control the spreading of the disease in China. Simply, the process for developing inactivated vaccine is as follows: *Chlamydia* seeds were isolated from the yolk sac membranes of 7-day-old chicken embryos between the 4 and 8th days after inoculation. The titer of *Chlamydia* used for vaccines was at least 10^12^ ELD_50_/0.4 ml. The yolk sac membrane was disrupted with a homogenizer and filtered through a mesh strainer. Formalin (0.3%) was used to inactivate *Chlamydia* with an equal volume of 206 adjuvant (SEPPIC, France) and mixed under 40 Mpa of pressure. After 7 days of inactivation with formalin, the safety and efficacy of the vaccine were tested using specific pathogen free (SPF) embryos and mice (100–102).

*Chlamydia* inactivated vaccines for sheep and goat have been used since 1981–1986 (100). During that time, 120,000 sheep and goats in Qinghai, Gansu province, and Xinjiang Uygur Autonomous region were vaccinated and the abortion rate declined sharply. No abortion happened due to vaccination in pregnant sheep and goats. A total of 2,000,000 ml (about 700,000 doses) of inactivated vaccine was produced and used in 1988 (100). The duration of immunity was at least 2 years, but 75% of the sheep and goats were protected from infection in the 4th year after vaccination (100, 103). Besides, the inactivated vaccine could be used after a 2 year storage period at 4°C (100). A similar inactivated vaccine of *Chlamydia* isolated from goat was studied by Zhang in the Inner Mongolia Autonomous Region (33). According to this report, 5,099 goats belonging to 51 groups were vaccinated with inactivated vaccine and at least 90% of the goats were protected. Other regions, such as Huachi County, Gansu province, showed similar results of very high prevalence of *Chlamydia* in goats during 1981–1986, with abortion rate in goats being 20–40%. The disease was controlled when inactivated vaccine was used, reducing the abortion rate of the vaccinated groups to 3.3–6.5%, compared with the control group abortion rate of 14.03–14.3%, during 1986–1988 (102).

*Chlamydia* of swine was also detected and isolated in Qinghai province and the GuangxiZhuang Autonomous Region (55, 56, 88). According to these reports, abortion happened among sows and the highest positive rate of abortion was 56.1% in one of the groups in which *Chlamydia* was detected using the complement fixation test (CFT). Two strains were isolated using 7-day-old SPF eggs, and the inactivated vaccine was produced and tested (101). A total of 1,080 sows were vaccinated in two farms and each sow was immunized subcutaneously with 3 ml of inactivated vaccine (101). After 3 months, 482 sows that had been vaccinated and 439 sows that were not vaccinated were studied; only 1.45% abortion rate was observed in the vaccinated group, while 29.53% abortion rate was observed in the non-vaccinated group (101). These results showed that the inactivated vaccine provided good infection protection (101). Subsequently, 10,594 sows were vaccinated in Qinghai, Shanxi province, and Guangxi Zhuang Autonomous regions. The duration of immunity was at least 1 year when 2 ml of vaccine was injected (104). The vaccine remained active after storage at 4–8°C for 1 year (101).

*Chlamydia* in bovines was reported firstly in China in 1986 (70). In 1990, 2 strains (CCS10 and CCS15) were isolated from cows in a farm in Shaanxi province (105). However, the strain used for the inactivated vaccine was isolated in 2006 (102). This isolated strain (SX5) from a farm in Shaanxi province was tested and the LD_50_/0.4 ml remained at 10^−12^ after at least 5 times propagation in SPF eggs. The minimal effective dosages for the vaccine was 3 ml for adult dairy cow and 1.5 ml for calf. The average protection was about 94.4%, while the duration of immunity was 10 months (102).

Although formalin-inactivated *C. abortus* vaccines have been used in China since 1985, their production was discontinued because of lack of good manufacturing practices (GMP), causing sporadic outbreaks in sheep (46), yaks (106), and other animals. However, there is no information about *Chlamydia* prevalence in recent years from Dulan county (100), Delingha (56), Qinghai province, and Huachi county (101), Gansu province, where *Chlamydia* was first isolated and animals were vaccinated with inactivated vaccine. On the contrary, farms near the original places, such as in Wulan county, Guinan county, Haiyan county, Gonghe county, Huzhu county, Huangyuan county, and Tianjun County, Qinghai province, reported that *Chlamydia* caused huge abortion among sheep, goats, and yaks in recent years (11, 35, 38, 68, 75, 84, 87, 107–109). Interestingly, during the investigation, shepherds reported that dead Tibetan antelopes were found and their eyes were obviously blind (personal communication). They also reported that *Chlamydia* spread among different groups of sheep that had never been in contact with each other, suggesting that wild animals may play a very important role to spread *Chlamydia* to domestic animal. Therefore, based on this information, we can conclude that the original source of *Chlamydia* infections in China is wild animals in Qinghai province.

Besides inactivated vaccine, the subunit vaccine for* Chlamydia* in ewes, which has three different doses of major outer-membrane protein from genetically engineered *Chlamydia psittaci,* was developed by the State Key Laboratory of Pathogen and Biosecurity, Institute of Microbiology and Epidemiology, Academy of Military Medical Sciences, China. The study analyzed the antibody responses in ewes vaccinated with the subunit vaccine of rCps-MOMP. The sera of ewes were detected before vaccination and at different times post-vaccination. Experimental results indicated that multilocus intramuscular injection in the neck region with a dose of 0.5 mg per ewe could stimulate good immune response (110). Because of the good security and immunity protection, a new veterinary drug certificate was awarded by the Ministry of Agriculture of the People's Republic of China (111). However, there are few reports of the promotion and application of the submit vaccine in China (112).

Although some successes were obtained for controlling *Chlamydia* in livestock in China since the inactivated vaccines were introduced, each year huge economic losses are caused by avian *Chlamydia* in chicken production (28). A subunit vaccine and a recombinant adenovirus live vector vaccine have been developed (16, 113). However, the recombinant adenovirus vector vaccine has not been approved by the government due to potential biological concerns, and no further data about the subunit vaccine has been published. Avian* Chlamydia* is still serious in China due to a lack of effective and safe vaccine. Moreover, the disease is a potential risk for human health. Therefore, further investigation into the development of vaccines is necessary.

## Treatment

Antibiotics such as tetracycline, oxytetracycline, and penicillin sodium are used to treat Chlamydiosis in animals in China. However, since 2014, to regulate the use of veterinary antibiotics in China, the government established a veterinary prescription drug management system, including measures for administration of veterinary prescription and over-the-counter drugs. The quality of antibacterial drug products has been improving every year. The quality of sampling inspection is maintained at more than 95%, whereas the rate of residues of veterinary drugs in livestock and poultry products remains stable at more than 97%. Thus, the use of antibiotics has been greatly reduced.

## Future Perspective

Although IHA plays a very important role in detecting *Chlamydia* in animals in China, it does not reflect the real situation of Chlamydiosis prevalence in animals. However, Hagemann JB (114) reported that aborting sheep exhibited a strong antibody response to surface (MOMP, MIP, and Pmp13G) and virulence-associated (CPAF, TARP, and SINC) antigens. While the latter disappeared within 18 weeks following abortion in majority of the animals, antibodies to surface proteins persisted beyond the duration of the study. In contrast, experimental non-abortion infected sheep developed antibodies mainly to surface antigens (MOMP, MIP, and Pmp13G), all of which did not persist. This indicates that new diagnostic methods need to be established to improve the accuracy of disease diagnosis and provides scientific basis for controlling animal Chlamydiosis.

## Conclusion

Since the first case of avian *Chlamydia* was reported in China, *Chlamydia* infection has been observed in different animals in most areas of China, which causes serious economic losses each year. Several diagnostic techniques, including CF, IHA, ELISA, ABC-ELISA, egg isolation, and PCR, have been studied and used in China. Formalin inactivated vaccines of* Chlamydia* from sheep, goat, swine, and cow were developed and have been used since 1981 in those areas where animals are threatened by *Chlamydia*. Because Chlamydiosis was considered an unimportant disease in animals by the Chinese government and no eradiation plan has implemented, there are sporadic outbreaks of *Chlamydia* in domestic animals in some areas, especially where vaccination has been suspended. However, the abortion rate was down sharply when inactivated vaccines for* Chlamydia* were used in domestic animals. This may have contributed to the lack of large-scale outbreak of Chlamydiosis in domestic animals in the last 30 years. The most important problem now is avian Chlamydiosis*,* which has a seropositive rate of 10–50% with IHA and easily spreads from birds to humans. Due to lack of effective vaccines, avian Chlamydiosis* may* become a public health problem in China.(Reorder)

## Author Contributions

Substantial contribution to the references of the work: ZLi, ZLou, YF. Draft and/or critical revision of the manuscript: JZ. Final approval of the version to be published: JZ, ZLi, ZLou, YF.

## Conflict of Interest Statement

The authors declare that the research was conducted in the absence of any commercial or financial relationships that could be construed as a potential conflict of interest.

## References

[1] Borel N Polkinghorne A Pospischil A A review on Chlamydial diseases in animals: still a challenge for pathologists? Vet Pathol (2018) 1:300985817751218. 10.1177/0300985817751218 29310550

[2] Dhama K Chakraborty S Tiwari R Singh SD Avian chlamydiosis (psittacosis / ornithosis): diagnosis, prevention and control, and its zoonotic concerns. Res Opin Anim Vet Sci (2013) 3(6):157–69.

[3] Campos-Hernández E Vázquez-Chagoyán JC Salem AZ Saltijeral-Oaxaca JA Escalante-Ochoa C López-Heydeck SM Prevalence and molecular identification of *Chlamydia abortus* in commercial dairy goat farms in a hot region in Mexico. Trop Anim Health Prod (2014) 46(6):919–24. 10.1007/s11250-014-0585-6 24715208

[4] Yang XL Zhang YX Xu DT Epidemiology investigationof *Chlamydia abortus* from sheep. Chinese J Vet Sci (1981) 7:13–15.

[5] Aaziz R Vorimore F Verheyden H Picot D Bertin C Ruettger A Detection of atypical Chlamydiaceae in roe deer (*Capreolus capreolus*). Vet Microbiol (2015) 181(3-4):318–22. 10.1016/j.vetmic.2015.10.018 26616600

[6] Szymańska-Czerwińska M Mitura A Niemczuk K Zaręba K Jodełko A Pluta A Dissemination and genetic diversity of chlamydial agents in Polish wildfowl: Isolation and molecular characterisation of avian *Chlamydia abortus* strains. PLoS One (2017) 12(3):e0174599 10.1371/journal.pone.0174599 28350846 PMC5370153

[7] Díaz JM Fernández G Prieto A Valverde S Lago N Díaz P Epidemiology of reproductive pathogens in semi-intensive lamb-producing flocks in North-West Spain: a comparative serological study. Vet J (2014) 200(2):335–8. 10.1016/j.tvjl.2014.02.022 24685472

[8] Yang R Jacobson C Gardner G Carmichael I Campbell AJ Ryan U Longitudinal prevalence and faecal shedding of Chlamydia pecorum in sheep. Vet J (2014) 201(3):322–6. 10.1016/j.tvjl.2014.05.037 24954870

[9] Longbottom D Entrican G Wheelhouse N Brough H Milne C Evaluation of the impact and control of enzootic abortion of ewes. Vet J (2013) 195(2):257–9. 10.1016/j.tvjl.2012.06.018 22809464

[10] Wilson K Sammin D Harmeyer S Nath M Livingstone M Longbottom D Seroprevalence of chlamydial infection in cattle in Ireland. Vet J (2012) 193(2):583–5. 10.1016/j.tvjl.2011.12.018 22285586

[11] Li Z Cao X Fu B Chao Y Cai J Zhou J Identification and characterization of *Chlamydia abortus* isolates from yaks in Qinghai, China. Biomed Res Int (2015) 2015:1–6. 10.1155/2015/658519 PMC4427853 26060818

[12] Vidal S Kegler K Greub G Aeby S Borel N Dagleish MP Neglected zoonotic agents in cattle abortion: tackling the difficult to grow bacteria. BMC Vet Res (2017) 13(1):373 10.1186/s12917-017-1294-y 29197401 PMC5712085

[13] Mcewen AD Foggie A Enzootic abortion in ewes: prolonged immunity following the injection of adjuvant vaccine. Vet Rec (1956) 68:686–90.

[14] Linklater KA Dyson DA Field studies on enzootic abortion of ewes in south east Scotland. Vet Rec (1979) 105(17):387–9. 10.1136/vr.105.17.387 552732

[15] Pan DX The research progress of *Chlamydia trachomatis* and *psittaci* in China. Chinese Journal Zoonoses (1987) 3(2):47–9.

[16] Zhou J Qiu C Cao XA Lin G Construction and immunogenicity of recombinant adenovirus expressing the major outer membrane protein (MOMP) of *Chlamydophila psittaci* in chicks. Vaccine (2007) 25(34):6367–72. 10.1016/j.vaccine.2007.06.031 17640776

[17] Shuai YY Huang MQ Wn G Cheng X Yang XL Study of *Chlamydia abortus* from sheep—pathogen isolation and identification. Chinese Journal Veterinary Science (1981) 8:1–5.

[18] Lenzko H Moog U Henning K Lederbach R Diller R Menge C High frequency of chlamydial co-infections in clinically healthy sheep flocks. BMC Vet Res (2011) 7:29 10.1186/1746-6148-7-29 21679409 PMC3125319

[19] Szymańska-Czerwińska M Niemczuk K Avian Chlamydiosis zoonotic disease. Vector Borne Zoonotic Dis (2016) 16(1):1–3. 10.1089/vbz.2015.1839 26741325

[20] Yu WJ Ma WL Zhu QT Yang XJ Wang WD Studies on epidemiology,clinical symptoms and pathogenesisof chicken Chlamydiosis. Chinese Journal Veterinary Science Technology (1994) 24(2):13–15.

[21] Shi Y He C Zhu H Duan Q Isolation and characterization of *Chlamydia psittaci* in broiler. Chinese Journal of Laboratory Animal Science (2003) 13(4):217–21.

[22] Ou YP Chen JJ Sun M He CL Shu G Ying ZQ Isolation and characterization of *Chlamydia psittaci* isolated from broilers. Chinese Veterinary Science (2016) 46(01):46–51.

[23] Wu FM Li YL Zhang JL Detection of avian Chlamydiosis. Chinese Journal of Zoonoses (1990) 6(1):61.

[24] Xu XP Ma YL Zheng AW Wang Y Fan QS Serological investigation of Chlamydia psittacifrom chicken in part of Sichuan areas. Sichuan Animal & Veterinary Sciences (1991) 01:18–19.

[25] Wang CY Wei YM Investigation of *Chlamydia* from sheep and chicken in Zhangye area, Gansu province. Chinese Journal of Animal Health Inspection (1991) 04:18.

[26] Wang QQ Liu BS Study on Chlamydiosis in chicks in Honghe district:isolation and identification of the causative agents and artificial infection test. Chinese Journal of Veterinary Science and Technology (1997) 27(3):10–12.

[27] Jiang SZ Zhang YQ Hu YJ Jiang YC Yao XJ Zhao YL Investigation of *Chlamydia psittaci* from chicken in two farms of Mudanjiang area. Heilongjiang Animal Science and Veterinary Medicine (1998) 07:25–6.

[28] Liu Q Li HM Guo JG Serological investigation of flow Chlamydiosis. Poultry Husbandry and Disease Control (2001) 11:13.

[29] Zhang JB Lu YK Cheng JH Serological investigation of pigeon Chlamydiosis. Chinese Journal of Veterinary Medicine (2003) 39(6):29.

[30] Ou CC Pan L Infection status investigation of *Chlamydia psittaci* from ducks in parts of Anhui area. China Poultry (2012) 02:61–3.

[31] Ma YJ Zhang LC Serological investigation of *Chlamydiosis* in livestock and poultry in Qinghai Province. Chinese Veterinary Science (2013) 43(02):213–5.

[32] Zhu YN Wang H Lei LC Epidemiological investigation of *Chlamydia infection* in Avian in Tianjin areas. Heilongjiang Animal Science and Veterinary Medicine (2016) 10:148–50.

[33] Zhang BF Zhao YF Zhou ZX Hu H Zhao MX Study on inactivated vaccine of *Chlamydia abortus* from goat. Chinese Journal of Animal and Poultry infections diseases (1987) 01:18–20.

[34] Li ZJ Wang QF Fang CX Chen MG Wu BR Wang YN Immunoprophylaxis of goat chlamydia abortion in Huachi County. Chinese veterinary technology (1990) 06:24–6.

[35] Su BH Study on serological of *Chlamydia* in sheep and cows in Gonghe County. Chinese Qinghai Journal of Animal and Veterinary Science (2016) 46(3):29–30.

[36] Wu LJ Liu Q Zheng LF Chen ZX Teng BZ Seroprevalence investigation of *Chlamydia abortus* from goats in Guangxi, China. Chinese Journal of Veterinary Science and Technology (2000) 30(9):41.

[37] Hong NN Yan XG Pi Q Hua Y Li JZ Serological survey for *Chlamydia* antibodies in cattle and goats in Guizhou Province. Progress in Veterinary Medicine (2012) 33(11):127–9.

[38] Zha XC Study on serological of *Chlamydia* in sheep and cows in Gonghe County. Shandong Journal of Animal Science and Veterinary Medicine (2017) 38(10):60–1.

[39] Wang XN Zhao GY Seroprevalence investigation of *Chlamydia abortusfrom* sheep in Zhejiang Province. Vet Sci China (1990) 20(8):11.

[40] Zhang XZ Zheng GS Gen XQ Investigation of Chlamydia abortus from sheep in XiangFan, Bubei province. Chinese Journal of Zoonses (1992) 8(1):34.

[41] Qiu CQ Zhou JZ Gao SD Cheng SM Duan YJ Li YC Monitoring of swine chlamydiosis on partial intensive pig farms in six Province. Chinese Journal of Veterinary Science and Technology (1998) 28(10):3–5.

[42] Wang QQ Zhang JW Zhang RH Investigation of Chlamydiosis from domestic animal in Honghe state, Yunnan Province. Chinese Journal of preventive veterinary medicine (2000) 22(6):465–6.

[43] Bao JM He CL Xu B Lv JM Ma Q Serological investigation of *Chlamydia abortus* from large scale breeding sheep in Ningxia Hui autonomous region. Gansu Animal and Veterinary Sciences (2003) 33(4):13–14.

[44] Wang L Sun CC Sui LY Tang KB Wang XF Wang GY Serological investigation of *Chlamydia abortus* from sheep in Hulunbeier, the NeiMonggol autonomous region. Animal Husbandry and Feed Science (2009) 31(1):154.

[45] Lei CH Guo FL Guo QY Shuan Z Lv CH Huang YB Serological investigation of *Chlamydia abortus* and *Mycoplasma* from sheep. Xinjiang Journal of Animal Husbandry (2012) 7:28–9.

[46] Huang SY Wu SM Xu MJ Zhou DH Danba C Gong G First record of *Chlamydia abortus* seroprevalence in Tibetan sheep in Tibet, China. Small Ruminant Research (2013) 112(1-3):243–5. 10.1016/j.smallrumres.2012.12.012

[47] Zhang LC Wang XZ Zhang MH La H You XQ Serological investigation of *Chlamydiosis* in livestock in Qinghai province. Chinese Journal of Veterinary Medicine (2014) 50(4):38–9.

[48] Cheng WW Peng JW Zhang KS Surveillance and analysis of *Chlamydia* seroprevalence from goat and sheep in some regions, China. Chinese Journal of Zoonnoses (2015) 31(5):472–4.

[49] Gao JY Hu JX Li C Detection and analysis of goat *Chlamydiosis* in part of Xinyang area in Henan province. Acta Ecologiae Animalis Domastici (2017) 38(1):66–9.

[50] Jiang TT Yang YS Meng QY Fang YL Han SL Serological investigation of *Chlamydia* from swine with HIA. Hubei Journal of Animal and Veterinary Sciences (1985) 6(4):32–4.

[51] Suo XF Wang DL Zhang CH The epidemiology investigation of serological from large scale swine farm in Hainan province. Swine Production (2005) 3:31–2.

[52] Bi JL Yang B Gao LB Yang GS Tai XY Yin GF Serological invetigation on porcine Choamydiae infection in Yunnan Province. Progress in Veterinary Medicine (2011) 3:134–6.

[53] Su YS Zhu LY Li PD Zhao Q Serological survey of porcine *Chlamydiae* infection in parts of Chuxiong prefecture in Yunnan Province. Journal of Anhui Agriculture Science (2015) 43(16):155–6.

[54] Ma CF Gao JY Hu JX Li H Ma Y Xu YJ Seral antibody detection of *Chlamydia* in breeding Swine herds in Xingyang of Henan province. Progress in Veterinary Medicine (2016) 37(10):119–22.

[55] Yi QZ Zheng LF Cheng ZX Zhou GJ Pang BH Liu Q Isolation and characterization of *Chlamydia abortus* from swine in Guang Xi Zhuang autonomous region. Guangxi Journal of Animal Husbandry & Veterinary Medicine (1991) 2:6–9.

[56] Diao YX Gl Y Zhao YX Li XF Ga QJ Jiao HZ Diagnosis and preventing of *Chlamydia abortus* in animal from Delinha farm, Qinghai province. Chinese journal of veterinary science and technology (1990) 3:21–2.

[57] Ji RG Fan WX Serological investigation of Chlamydia in swine. Shandong Journal of Animal Husbandry and Veterinary Science (2003) 24(1):39.

[58] Gao SZ Wei RS Shi YN Shu Z Han QY He HC Serological investigation of Chlamydia from swine in Gansu Province. Gansu Animal and Veterinary Sciences (2001) 33(2):13–14.

[59] Lang LM Wang KL Xiong HD You G Zhang B Serological investigation of Chlamydia in infected swine in Henan Province. Swine Production (2004) 19(4):29.

[60] Wang HZ Serological investigation of *Chlamydia* from swine of large scale breeding farm. Modern Journal of Animal Husbandry and Veterinary Medicine (2004) 33(11):26.

[61] Jin AH Gu BL Wei XY Fang GM Serological investigation of Chlamydia from swine in Shanghai and surrounding areas. Shanghai Journal of Animal Husbandry and Veterinary Medicine (2005) 50(6):23.

[62] Zhu YQ Wang RB Cai YQ Hu JC Lai XX Serological investigation of Chlamydiosis from swine. Guangdong Journal of Animal and Veterinary Science (2007) 32(4):26–7.

[63] Zhou P Lin ZY Wang TF Luo CQ Qiu SS Zhang HQ Investigation on sera epidemiolog of porcine chlamydiosis in several regions of Longyan city. Progress in Veterinary Medicine (2008) 29(7):30–5.

[64] Wang JQ Wei H Wang WH Ma NJ Seroprevalence investigation of reproductive obstacle disease from sow. Contemporary Animal Husbandry (2013) 41(15):9–10.

[65] Jiang HH Serological investigation of *Toxoplasmosis* and *Chlamydiosis* in pigs in Jiangxi Province and genotyping of *Toxoplasma gondii* from pigs from different localities in China. The Chinese Academy of Agricultural Sciences (2014):27–8.

[66] Li YL Serological survey of porcine *Chlamydiae* infection in parts of Chuxiong prefecture in Yunnan Province. Veterinary science and technology (2014) 33:29–30.

[67] Zhou JZ Qiu CQ Cheng SM Gao SD Serological study of *Chlamydiosis* in meat cattle at the partial areas in China. Chinese Journal of Veterinary Science and Technology (2000) 30(7):14–15.

[68] Xie Q Zeng J Zhao XR Wu E Wang N The *Chlamydia abortus* isolation and identification from cow in Ning Xia Hui autonomous region, China. Heilongjiang Animal Science and Veterinary Medicine (2012) 05:102–4.

[69] Wang WY Xie AM Lin YQ Shen YL Cai JS Study on serological of *Chlamydia* in cows in Huzhu County. Chinese Qinghai Journal of Animal and Veterinary Science (2016) 46(3):16–17.

[70] Yang YS Jiang TT Fang YL Meng QY Tan HY Yong Z Diagnosis report of mixed infection with cow Chlamydiosis and brucellosis. Hubei Journal of Animal and Veterinary Sciences (1986) 04:4–9.

[71] Li F Zhang DM Liang ZM Serological investigation of *Chlamydiosis* of cows in partial dairies in Henan Province. Journal of Agricultural Catastrophology (2017) 7(2):22–4.

[72] Yang YS Jiang TT Fang YL Meng QY Tan HY Yong Z Diagnosis report of mixed infection with cow Chlamydiosis and brucellosis. Chinese Journal of Veterinary Medicine (1988) 14(9):5–6.

[73] Huo BN Serological investigation and control of *Chlamydia abortus* and *Brucella* from cows. Livestock and Poultry Industry (2013) 24(6):67.

[74] Tan QD LI ZX Wang XL Yin MY Qin SY Liu GX Seroprevalence investigation and risk ractors analysis of Chlamydia infection in dairy cattle in Gansu and Ningxia areas. Vet Med (2015) 42(5):1283–7.

[75] Chen CJ Fan XL Tan ML Serological survey and analysis on *Brucella*, *Chlamydia*, and *Toxoplasmosis* on scale dairy farming in Huangyuan county of Qinghai. Chinese Qinghai Journal of Animal and Veterinary Science (2017) 47(1):33–5.

[76] Shuai YY Yc L Dan K Duan YJ Cheng GL Yang ZP Diagnosis of enzootic abortion in yak. Scientia Agriculture Sinica (1988) 21(4):76–81.

[77] Wang GH Shi BX Ma XG Serological investigation of *Chlamydiosis* in Yaks. Grass-feeding Livestock, Grass-feed Lives (1996) 03:46.

[78] Hou SK Serological investigation of *Chlamydiosis* in Yaks in Guoluo areas of Qinghai province. Chinese Qinghai Journal of Animal and Veterinary Science (2011) 41(04):10.

[79] Qin SY Huang SY Yin MY Tan QD Liu GX Zhou DH Seroprevalence and risk factors of *Chlamydia abortus* infection in free-ranging white yaks in China. BMC Vet Res (2015) 11:8 10.1186/s12917-015-0323-y 25601354 PMC4308933

[80] Dong CX Bu D Ban JM Serological investigation of *Chlamydiosis* in Yaks in Guoluo areas of Qinghai province. Chinese Qinghai Journal of Animal and Veterinary Science (1993) 02:25.

[81] Ma SL Li XH Lu Y Yang QY Pen XX Ma DK Serological investigation of *Chlamydiosis* in Yaks in Qinghai province. Chinese Qinghai Journal of Animal and Veterinary Science (2004) 34(5):14.

[82] Zhang XQ Li WC Wan MDZ Lu Y Investigation and analysis of *Chlamydiosis* in Yaks in Tianjun areas of Qinghai province. Gansu Animal Science and Veterinary Medicine (2009) 39(03):14–15.

[83] Kong XY Bao SK He CJ Serological investigation of *Chlamydiosis* and *Brucella* in Yaks in Qinghai Lake areas. Animals Breeding and Feed (2012) 11:51–2.

[84] Li JH Zhang XQ Li WC Zhou JZ Serological investigation of *Chlamydiosis* and *Brucella* in Yaks in Tianjun areas of Qinghai province. Animal Husbandry & Veterinary Medicine (2013) 45(7):126.

[85] Xie ZX Epidemic dynamic investigation of Chlamydiosis in Yaks in Guoluo areas of Qinghai province. Chinese Qinghai Journal of Animal and Veterinary Science (2013) 43(1):33.

[86] Yin MY Tan QD Qin SY Liu GX Zhu XQ Zhou DH Seroprevalence of *Chlamydia* Infection in Yaks in Gansu Province. Journal of Animal Science and Veterinary Medicine (2014) 41(10):281–5.

[87] Fu YG Chao YL Chang MH Investigation and analysis of *Chlamydiosis* in yaks and Tibetan sheep in Qinghai province. Chinese Journal of Veterinary Medicine (2016) 52(9):50–1.

[88] Yang YS Jiang TT Men QY Studies on indirect hemagglutinationtest (IHA) to determine against Chlamydia antibodies Chinese. Journal of Animal and Veterinary Sciences (1984) 15(3):181–7.

[89] Ge WN Shuai YY Dan K Li YC Yang ZP Yang XL Study on antibody of *Chlamydia **abortus* detected by ELISA. Chinese Journal of Veterinary Science and technology (1986) 04:16–19.

[90] Yang ZP An ABC-ELISA for detecting antibodies against abortigenic Chlamydiae in sheep and goats. Chinese Journal of Zoonses (1990) 2:38–41.

[91] Yang X Shuai YY Ge WN Huang MQ Li YC Cheng ZT Isolation of *Chlamydia abortus* from sheep. Chinese Journal of Veterinary Science and Technology (1984) 2:83–9.

[92] Okuda H Ohya K Shiota Y Kato H Fukushi H Detection of *Chlamydophila psittaci* by using SYBR green real-time PCR. J Vet Med Sci (2011) 73(2):249–54. 10.1292/jvms.10-0222 20948172

[93] Wittenbrink MM Schoon HA Schoon D Mansfeld R Bisping W Endometritis in cattle experimentally induced by *Chlamydia psittaci*. Zentralblatt Veterinarmedizin Reihe B (1993) 40(6):437–50. 10.1111/j.1439-0450.1993.tb00161.x 8284957

[94] Kaltenboeck B Kousoulas KG Storz J Two-step polymerase chain reactions and restriction endonuclease analyses detect and differentiate ompA DNA of *Chlamydia* spp. J Clin Microbiol (1992) 30(5):1098–104. 1349899 10.1128/jcm.30.5.1098-1104.1992 PMC265232

[95] Gutierrez J O’Donovan J Proctor A Brady C Marques PX Worrall S The application of the policy real-time polymerase chain reaction for the diagnosis of toxoplasmosis and enzootic abortion of ewes. J Vet Diagn In Vest (2012) 24(5):846–54. 10.1177/1040638712452730 22807509

[96] Thiele D Wittenbrink MM Fischer D Krauss H Evaluation of the polymerase chain reaction (PCR) for detection of *Chlamydia psittaci* in abortion material from ewes. Zentralbl Bakteriol (1992) 277(4):446–53. 10.1016/S0934-8840(11)80469-X 1303688

[97] Mi ZH Qin L Study on detection methods of *Chlamydia psittaci* by nested PCR and DNA sequencing analysis. Chinese Journal of zoonoses (2002) 18(3):68–70.

[98] Fei YY Li ZC Lou ZZ Detection of Chlamydia in livestock by recombine polymerase amplification. Chinese Veterinary Science (2018) 48(02):142–7.

[99] Chen Q Gong X Zheng F Cao X Li Z Zhou J Seroprevalence of *Chlamydophila abortus* infection in yaks (*Bos grunniens*) in Qinghai, China. Trop Anim Health Prod (2014) 46(3):503–7. 10.1007/s11250-013-0519-8 24343703

[100] Shuai YY Li YC Yang ZP Dan K Cheng GL Duan YJ Study on yolk sac membrane methanol killing and oil adjuvant vaccine of *Chlamydia abortus* in sheep. Chinese journal of veterinary science and technology (1989) 11:3–6.

[101] Li ZJ Wang QF Fang CX Chen MG Wu BR Wang YN Immunization test of *Chlamydia abortus* vaccine from goats in Huachi County, China. Chinese journal of veterinary science and technology (1990) 06:24–6.

[102] Qiu CQ Zhou JZ Cheng SM Cao XA Identification and immunogenicity of pathogen causing Chlamydial abortion in dairy cows. Vet Sci China (2006) 04:270–3.

[103] Foggie A The duration of immunity in ewes following vaccination against ovine enzootic abortion virus. Vet Rec (1959) 71:741–2.

[104] Li YC Shuai YY Duan YJ Gao SD Yang XL Qiu CQ Study on inactivated vaccine of *Chlamydia abortus* in pigs. Chinese journal of veterinary science and technology (1995) 25(11):3–7.

[105] Lin ZY Huang MR Zhang ZZ Liang DJ Li YC Duan YJ The isolation and identification of *Chlamydia abortus* in cow. Chinese journal of veterinary science and technology (1992) 02:27–30.

[106] Gao LY Zhang DH Wang QJ Pu ZZ Chen DT The seroprvevalence investigation of Chlamydia in yaks in Tianjun County. China Shanghai Journal of Animal Husbandry and Veterinary Medicine (2013) 01:3.

[107] Zhang XQ Li WC Seroprevalence investigation of *Chlamydia abortus* in Tibetan sheep in Tianjun County, Qinghai province. Chinese Journal of Veterinary Medicine (2011) 10:45.

[108] Zhang YL Li CH Seroprevalence investigation of *Chlamydia* in yaks and sheep in HaiYan County, China. Heilongjiang Animal Science and Veterinary Medicine (2011) 6:79–80.

[109] Zhang YL Seroprevalence investigation of *Chlamydia* in yaks and sheep in WuLan County, Qinghai province. Heilongjiang Animal Science and Veterinary Medicine (2012) 2:88–9.

[110] Li YW Yang BF Song LH M GH Zhu H Duan Q Kinetics curve of antibody responses in ewes immunized with subunit vaccine of genetically engineered *Chlamydia psittaci*. Chinese Journal of Zoonoses (2010) 26(9):835–7.

[111] Zhu WG Zheng J Lianx Z Cui J Xie Q The research progress of animal chlamydia vaccine. Modern Journal of Animal Husbandry and Veterinary Medicine (2015) 9:41–5.

[112] Zhang ZJ Zhou JZ Progress on *Chlamydia abortus*. Progress in Veterinary Medicine (2017) 38(4):102–7.

[113] He C Zhu H Wang CW He J Zhang HJ Tan H Investigation on immune response in broiler immunized with recombinant major outer-membrane protein of *Chlamydia psittaci*. J China Univ Geosci (2004) 01:45–8.

[114] Hagemann JB Simnacher U Longbottom D Livingstone M Maile J Soutschek E Analysis of humoral immune responses to surface and virulence-associated *Chlamydia abortus* proteins in ovine and human abortions by use of a newly developed line immunoassay. J Clin Microbiol (2016) 54(7):1883–90. 10.1128/JCM.00351-16 27194684 PMC4922118

